# This Is the Year That Was: The Best of the Biological Sciences section of The Journals of Gerontology Series A

**DOI:** 10.1093/geroni/igaf122.2052

**Published:** 2025-12-31

**Authors:** Gustavo Duque, Fabrisia Ambrosio

## Abstract

Following the tradition started in 2023, this symposium will present the best papers published in 2024 in the Biological Sciences section of The Journals of Gerontology, Series A: Biological Sciences and Medical Sciences. This will be a great opportunity for the authors of the top ranked papers to interact with the audience, receive feedback and answer to their questions in an interactive manner.

